# Long-Term Tolerability, Safety, and Efficacy of Recombinant Human Hyaluronidase-Facilitated Subcutaneous Infusion of Human Immunoglobulin for Primary Immunodeficiency

**DOI:** 10.1007/s10875-016-0298-x

**Published:** 2016-05-25

**Authors:** Richard L. Wasserman, Isaac Melamed, Mark R. Stein, Werner Engl, Marlies Sharkhawy, Heinz Leibl, Jennifer Puck, Arye Rubinstein, Lisa Kobrynski, Sudhir Gupta, Andrew J. Grant, Anoshie Ratnayake, Wendell G. Richmond, Joseph Church, Leman Yel, David Gelmont

**Affiliations:** Allergy Partners of North Texas Research, Dallas, TX USA; IMMUNOe Clinical Research Center, Centennial, CO USA; Allergy Associates of the Palm Beaches, North Palm Beach, FL USA; Baxalta Innovations GmbH, Vienna, Austria; University of California San Francisco, San Francisco, CA USA; Allergy & Immunology Division, Montefiore Medical Center, Bronx, NY USA; Emory Children’s Center, Emory University, Atlanta, GA USA; University of California, Irvine, CA USA; University of Texas Medical Branch, Galveston, TX USA; West Coast Clinical Trials, Cypress, CA USA; Allergy and Asthma Physicians, Hinsdale, IL USA; Children’s Hospital Los Angeles, Los Angeles, CA USA; Baxalta US Inc., Cambridge, MA USA; Baxalta US Inc., Westlake Village, CA 91362-3811 USA

**Keywords:** Subcutaneous IgG replacement, recombinant human hyaluronidase, primary immunodeficiency, efficacy, tolerability

## Abstract

**Purpose:**

Treatment of primary immunodeficiency diseases (PIDD) with subcutaneous (SC) infusions of IgG preceded by injection of recombinant human hyaluronidase (rHuPH20) (IGHy) to increase SC tissue permeability was evaluated in two consecutive, prospective, non-controlled, multi-center studies.

**Methods:**

Subjects >4 years of age received SC IgG replacement at a weekly dose equivalent of 108 % of their previous intravenous (IV) dose, facilitated by prior injection of 75 U/g IgG of rHuPH20. Starting with weekly SC infusions, the interval was increased (ramped-up) to a 3- or 4-week schedule.

**Results:**

Eighty-three subjects (24 < 18 years; 59 ≥ 18 years) received 2729 infusions (excluding ramp-up) at a mean dose of 0.155 g/kg/week in the pivotal and 0.156 g/kg/week in the extension study. IGHy exposure exceeded 30 months in 48 subjects.

During 187.7 subject-years of IGHy exposure, 2005 adverse events (AEs) (10.68 per subject-year) occurred. The rate of related systemic AEs during consecutive 1-year periods remained low; the rate of related local AEs decreased from 3.68/subject-year in months 1–12 to approximately 1.50/subject-year after 30 months of treatment. Fifteen subjects transiently developed anti-rHuPH20 binding antibody. There was no difference in AE rates in these subjects before and after the first titer increase to ≥1:160.

The rate of infections during IGHy exposure was 2.99 per subject-year and did not increase during the studies. Annual infection rates were 3.02 in subjects <18 years and 2.98 in subjects ≥18 years.

**Conclusions:**

Long-term replacement therapy with IGHy was safe and effective in 83 pediatric and adult subjects with PIDD.

**Electronic supplementary material:**

The online version of this article (doi:10.1007/s10875-016-0298-x) contains supplementary material, which is available to authorized users.

## Introduction

Subcutaneous (SC) immunoglobulin (IgG) replacement therapy in patients with primary immunodeficiency diseases (PIDD) has been shown to be as efficacious as intravenous (IV) treatment while causing fewer systemic adverse reactions [1–4]. SC infusion proved to be beneficial specifically in patients at risk of systemic reactions but also in patients, including infants, in whom stable venous access is difficult to maintain [3–10]. Because the incidence of systemic adverse reactions is low and venous access is not required, self-infusion of IgG via the SC route can be performed by patients at home providing greater ease and convenience compared to IV administration in a hospital or infusion center [3, 11–15].

The main disadvantages of SC therapy have been the limited volume that can be infused in a single SC site and the lower bioavailability of IgG after SC compared to IV administration, necessitating the use of multiple infusion sites on a weekly or every-other-week basis and an increased dose compared to IV infusion in order to provide the same exposure as measured by the area under the time-concentration curve [16, 17].

Hyaluronan (hyaluronic acid), the main component of the SC extracellular matrix (ECM), causes resistance to bulk fluid flow through the SC tissue. Cleavage of hyaluronan by subcutaneously injected hyaluronidase, a highly specific glycosidase, increases the permeability of SC tissue. In the SC space, hyaluronan is rapidly resynthesized, and the interstitial viscosity is fully restored within 24 to 48 h [18]. Recombinant human hyaluronidase (rHuPH20), a highly purified soluble form of a naturally occurring human hyaluronidase suitable for chronic use in humans, is safe and effective in enhancing dispersion and absorption of fluids and drugs administered subcutaneously [19–23]. Preclinical studies showed that rHuPH20 is short-acting, with a half-life of <30 min, and is undetectable in plasma after administration at the doses used to facilitate SC infusions [18, 23].

A recent pivotal study in 83 subjects with PIDD demonstrated that pre-infusion of rHuPH20 allowed SC administration of large volumes of IgG in a single infusion site every 3–4 weeks, comparable to an IV treatment schedule. SC infusion of IgG facilitated by rHuPH20 (IGHy) was safe, effective, and well tolerated despite high infusion volumes and rates [23]. Results after extended IGHy replacement therapy in the pivotal and an extension study are reported here.

## Methods

### Study Design

Long-term safety, tolerability, and efficacy of IGHy treatment in PIDD were evaluated in subjects participating in two consecutive, phase 3, prospective, open-label, non-controlled, multi-center studies. The studies were performed in accordance with the International Conference on Harmonization Good Clinical Practice (ICH GCP) and applicable legal requirements and registered on ClinicalTrials.gov (NCT00814320 and NCT01175213). The study protocols and informed consent forms were reviewed and approved by the appropriate ethics committees. Written informed consent was obtained from all subjects and/or their legally authorized representatives prior to performing any study-related procedures. Assent was obtained when appropriate.

### Treatment

A 10 % preparation of normal human immunoglobulin stabilized with glycine (GAMMAGARD LIQUID in the USA/Canada; elsewhere KIOVIG; Baxalta US Inc., Westlake Village, CA) was administered intravenously (referred to as immune globulin intravenous [IGIV]) and subcutaneously (immune globulin subcutaneous [IGSC]) in combination with rHuPH20 (IGHy). rHuPH20 (Halozyme Therapeutics, Inc., San Diego, CA) component of IGHy is a preparation of purified recombinant soluble human hyaluronidase produced in Chinese hamster ovary cells formulated at a concentration of 160 U/mL in a buffer solution containing 1 % human albumin.

The pivotal study comprised two epochs: In epoch 1, subjects received IGIV at their pre-study dose and interval for 3 months to determine pharmacokinetics of IGIV treatment. Subjects who had participated in a previous study which comprised a 3-month period of IGIV treatment followed by 1 year of IGSC treatment could immediately enter epoch 2, as pharmacokinetics of IGIV treatment were already known from the previous study [3]. For epoch 2 infusions, rHuPH20 at a dose of 75 U/g IgG was administered through a 24-gauge SC needle, followed by IGSC at 108 % of the weekly IGIV dose equivalent, via the same SC needle. Ramp-up to allow adaptation to large SC doses began in epoch 2 with an initial 1-week dose (25 % of the monthly dose) of IGHy and increased until the full dose at the pre-study IGIV interval (3 or 4 weeks) was reached [23]. After approximately 14 to 18 months of IGHy treatment in epoch 2, subjects could enter the extension study. In that study, IGHy dose and infusion interval were maintained for the first three infusions. Thereafter, subjects had the option to switch from a 3- or 4-week to a 2-week treatment interval using one half the calculated 4-week dose for a maximum of 4 months to allow evaluation of trough levels for the 2-week SC interval. In a final safety follow-up, subjects changed to IGIV or IGSC alone and anti-rHuPH20 antibody titers were monitored for up to 48 weeks.

### Study Population

Patients aged >2 years with PIDD involving an antibody production defect and requiring antibody replacement as defined by the International Union of Immunological Societies [24, 25] were eligible for the pivotal study if they had been receiving IgG for >3 months before enrollment at a dose of ≥300 mg/kg of body weight/4 weeks. Completion of the pivotal study was a prerequisite for inclusion in the extension study.

### Endpoints

Efficacy was assessed during IGHy dosing in the pivotal and extension studies. The primary objective was the rate of validated acute serious bacterial infections (VASBIs) per year. Additional efficacy endpoints included the rate of any infection, days off school/work, days on antibiotics, number of non-study out-patient visits, number of hospitalizations, and days in hospital.

Safety and tolerability were monitored during the combined pivotal and extension study periods lasting up to 3.5 years. Endpoints assessed included the annual rate of adverse events (AEs); rates of AEs by subject and by infusion; categorization of AEs by seriousness, severity, causality, and temporal association with study treatment.

Study subjects were monitored for the development of rHuPH20-reactive binding or neutralizing antibodies, and an association between antibody formation and clinical or laboratory AEs was assessed.

In the extension study, the effect of varying IGHy infusion frequency on IgG trough levels was assessed. Specific antibodies to relevant pathogens at the end of IGIV treatment in the pivotal study and at the end of IGHy treatment in the extension study were determined.

An exploratory endpoint of treatment preference was studied by surveying subjects at completion of IGHy treatment.

### Statistical Analysis

Analyses included the period from the first administration through the end of IGHy treatment. Numbers and rates of all, local, systemic, related and temporally associated AEs and of infections were calculated per subject per year for the entire study population and stratified for <18 and ≥18 years. Analyses in 1-year periods over time included only subjects who received IGHy treatment for the full 1-year period.

Trough levels of IgG at the end of IGHy infusion cycles were analyzed in relation to dose frequency. Median trough levels and their non-parametric 95 % confidence intervals were calculated for 2-, 3-, and 4-week infusion intervals and for age groups (<18 and ≥18).

Point estimates and 95 % CI were calculated for the annual rates of days off work/school, days on antibiotics, number of non-study out-patient visits, hospitalizations, and days in hospital.

## Results

### Subjects and Exposure

**Fig. 1:**
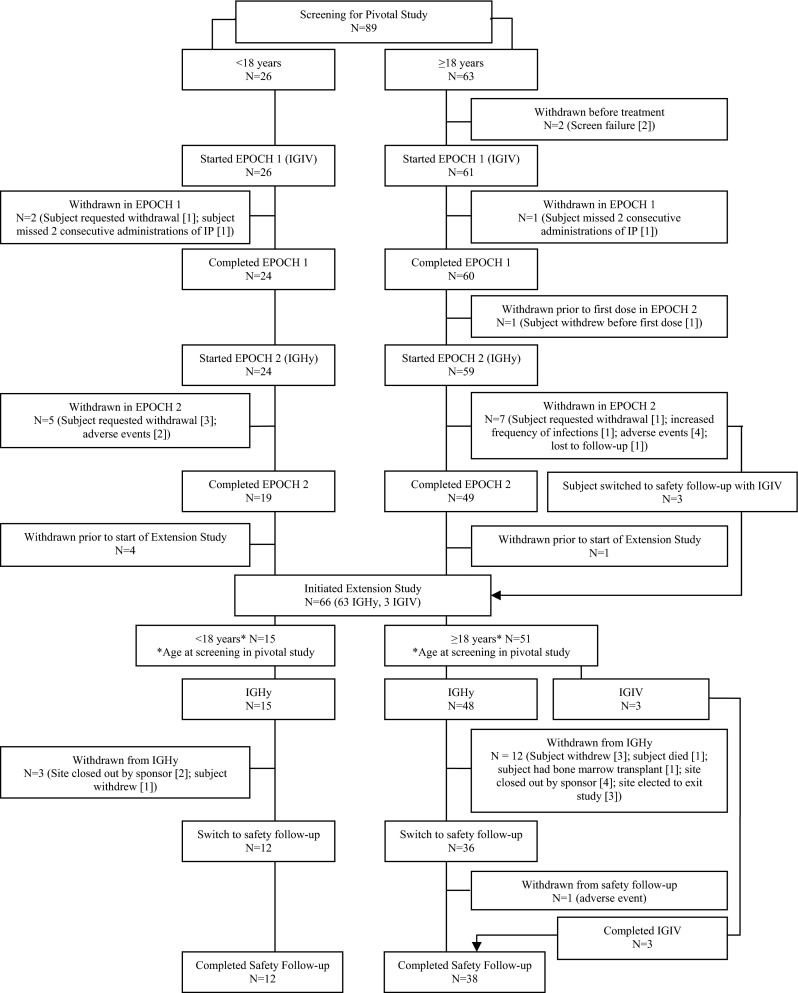
Disposition of subjects

A total of 89 subjects, 46 male and 43 female, at 14 sites in the USA and Canada enrolled in the pivotal study. Age at enrollment ranged from 4 to 78 years. Eighty-seven (87) subjects received IGIV for a 3-month period either in epoch 1 (*n* = 56) or during a previous study (*n* = 31). Eighty-three (83) subjects (24 < 18 years and 59 ≥ 18 years) continued on to epoch 2 of IGHy treatment. Sixty-six (66) subjects at 11 sites rolled over into the extension study: 63 continued on IGHy and 3 on IGIV treatment. For details of subject demographics and disposition including age groups, refer to the online supplementary material Table E1 and Fig. 1.

In the pivotal study, 365 IGIV infusions were administered in epoch 1 and 1359 infusions of IGHy were administered in epoch 2: 230 during and 1129 after ramp-up [23]. Subjects in the extension study received 1600 IGHy infusions. Table 1 shows the exposure to IGHy, excluding ramp-up, by age group. A mean ± SD IGSC dose of 0.155 ± 0.053 g/kg/week (excluding ramp-up) was administered in the pivotal study resulting in a mean volume of 292.2 mL per infusion site [23]. The mean ± SD IGSC dose was similar in the extension study at 0.156 ± 0.051 g/kg/week (data not shown). Infusions were administered at a median maximum rate of 300 mL/h [23].

**Table 1:** IGHy infusions during pivotal (excluding ramp-up) and extension studies by age group (<18, ≥18 years)

	Pivotal study (excluding ramp-up)		Extension study	
Age group^a^ (years)	Number of subjects treated	Number of infusions administered	Number of subjects treated	Number of infusions administered
<18	22	310	15	364
≥18	59	819	48	1236
Total	81	1129	63	1600

^a^Age at screening in pivotal study

Including ramp-up, subjects experienced IGHy treatment for a total of 187.7 subject-years (online supplementary material Table E5). The mean ± SD duration of IGHy treatment was 367.7 ± 103.9 days in the pivotal study (excluding ramp-up) and 565.9 ± 211.8 days in the extension study (data not shown). For 48 subjects, IGHy exposure exceeded 30 months (Table 4).

### Safety

A total of 2005 AEs (excluding infections) were reported during IGHy exposure. Among 498 local AEs (2.65 per subject-year), 488 (2.60 per subject-year) occurred during or within 72 h after infusion; 488 (2.60 per subject-year) were considered related to IGHy treatment by the investigator. Subjects <18 years experienced fewer related local AEs (1.38 per subject-year) than subjects ≥18 years (3.03 per subject-year). A total of 1507 systemic AEs (8.03 per subject-year), excluding infections, were reported during IGHy exposure: 491 (2.62 per subject-year) occurred during or within 72 h after treatment; 329 systemic AEs (1.75 per subject-year) were considered related to IGHy treatment by the investigator (online supplementary material Table E2).

The rate of related systemic AEs over 1-year periods remained consistently low while the rate of related local AEs gradually decreased from 3.68 to 1.51 per subject year after 30 months of IGHy treatment (online supplementary material Table E3).

During IGHy treatment, local AEs (including infections) occurred at a rate of 0.17 per infusion. Overall, differences in the frequency and severity of local AEs among body mass index (BMI) groups were small. Rates of local AEs/infusion were 0.13 in subjects with a BMI <25, 0.19 in subjects with a BMI of 25 to 30, and 0.23 in subjects with a BMI >30. The majority of local AEs, irrespective of BMI, were mild (398/500, 0.14 per infusion). In subjects with a BMI <25 (*N* = 48), 47/205 local AEs (0.03 per infusion) were moderate and 2/205 (0.00 per infusion) were severe. In subjects with a BMI between 25 and 30 (*N* = 17), 12/127 local AEs (0.02 per infusion) were moderate and none was severe, and in subjects with a BMI >30 (*N* = 18), 39/168 local AEs (0.05 per infusion) were moderate and 2/168 AEs (0.00 per infusion) were severe (online supplementary material Table E4).

A total of 23 (0.12 per subject-year) serious systemic adverse events (SAEs) occurred, none of which was related to IGHy treatment (online supplementary material Table E5): 5 (0.10 per subject-year) in the <18 years and 18 (0.13 per subject-year) in the ≥18-year age group.

During IGHy exposure, the AEs reported by most subjects were headache (54.2 %), followed by infusion site pain (53.0 %), nausea (32.5 %), vomiting and asthma (28.9 % each), fatigue (26.5 %), infusion site erythema and pyrexia (25.3 % each), diarrhea (24.1 %), and arthralgia and cough (19.3 % each). All AEs occurring in ≥5 % of subjects are listed in Table 2. No serious hypersensitivity reactions attributable to IGHy occurred during long-term treatment.

**Table 2:** All AEs (including infections) reported in ≥5 % of subjects during IGHy treatment (including ramp-up)

		AEs	By subject *N* = 83	By subject-year *N* = 187.69	By infusion *N* = 2959
System organ class	Preferred term	*n*	*n* (%)	*n* (rate)^a^	*n* (%)^b^
Blood and lymphatic system disorders	Lymphadenopathy	14	10 (12.0 %)	14 (0.07)	14 (0.47 %)
Cardiac disorders	Tachycardia	10	8 (9.6 %)	10 (0.05)	10 (0.34 %)
Gastrointestinal disorders	Nausea	74	27 (32.5 %)	74 (0.39)	70 (2.37 %)
	Vomiting	38	24 (28.9 %)	38 (0.20)	38 (1.28 %)
	Diarrhea	34	20 (24.1 %)	34 (0.18)	33 (1.12 %)
	Upper abdominal pain	19	12 (14.5 %)	19 (0.10)	19 (0.64 %)
	Abdominal pain	17	10 (12.0 %)	17 (0.09)	14 (0.47 %)
	Constipation	9	9 (10.8 %)	9 (0.05)	9 (0.30 %)
	Gastroesophageal reflux disease	6	6 (7.2 %)	6 (0.03)	6 (0.20 %)
	Aphthous stomatitis	9	5 (6.0 %)	9 (0.05)	9 (0.30 %)
	Dental caries	7	5 (6.0 %)	7 (0.04)	7 (0.24 %)
	Hemorrhoids	6	5 (6.0 %)	6 (0.03)	6 (0.20 %)
General disorders and administrative site conditions	Infusion site pain	213	44 (53.0 %)	213 (1.13)	202 (6.83 %)
	Fatigue	52	22 (26.5 %)	52 (0.28)	51 (1.72 %)
	Infusion site erythema	70	21 (25.3 %)	70 (0.37)	70 (2.37 %)
	Pyrexia	42	21 (25.3 %)	42 (0.22)	40 (1.35 %)
	Infusion site discomfort	41	11 (13.3 %)	41 (0.22)	39 (1.32 %)
	Pain	22	11 (13.3 %)	22 (0.12)	22 (0.74 %)
	Infusion site pruritus	52	8 (9.6 %)	52 (0.28)	51 (1.72 %)
	Infusion site swelling	17	8 (9.6 %)	17 (0.09)	15 (0.51 %)
	Asthenia	20	7 (8.4 %)	20 (0.11)	19 (0.64 %)
	Infusion site edema	17	7 (8.4 %)	17 (0.09)	17 (0.57 %)
	Local swelling	9	7 (8.4 %)	9 (0.05)	9 (0.30 %)
	Peripheral edema	15	7 (8.4 %)	15 (0.08)	14 (0.47 %)
	Chest pain	5	5 (6.0 %)	5 (0.03)	5 (0.17 %)
Infections and infestations	Sinusitis	122	47 (56.6 %)	122 (0.65)	122 (4.12 %)
	Upper respiratory tract infection	78	41 (49.4 %)	78 (0.42)	74 (2.50 %)
	Viral upper respiratory tract infection	41	18 (21.7 %)	41 (0.22)	41 (1.39 %)
	Bronchitis	34	17 (20.5 %)	34 (0.18)	34 (1.15 %)
	Viral gastroenteritis	19	15 (18.1 %)	19 (0.10)	19 (0.64 %)
	Viral infection	13	12 (14.5 %)	13 (0.07)	13 (0.44 %)
	Influenza	13	11 (13.3 %)	13 (0.07)	13 (0.44 %)
	Nasopharyngitis	18	11 (13.3 %)	18 (0.10)	18 (0.61 %)
	Chronic sinusitis	14	10 (12.0 %)	14 (0.07)	14 (0.47 %)
	Gastroenteritis	11	10 (12.0 %)	11 (0.06)	11 (0.37 %)
	Urinary tract infection	17	10 (12.0 %)	17 (0.09)	17 (0.57 %)
	Cellulitis	9	9 (10.8 %)	9 (0.05)	9 (0.30 %)
	Oral herpes	10	8 (9.6 %)	10 (0.05)	10 (0.34 %)
	Pharyngitis	10	8 (9.6 %)	10 (0.05)	10 (0.34 %)
	Post procedural infection	7	7 (8.4 %)	7 (0.04)	7 (0.24 %)
	Ear infection	8	6 (7.2 %)	8 (0.04)	7 (0.24 %)
	Acute sinusitis	6	5 (6.0 %)	6 (0.03)	6 (0.20 %)
	Pneumonia	5	5 (6.0 %)	5 (0.03)	5 (0.17 %)
	Respiratory tract infection	6	5 (6.0 %)	6 (0.03)	5 (0.17 %)
Injury, poisoning, and procedural complications	Procedural pain	14	12 (14.5 %)	14 (0.07)	14 (0.47 %)
	Excoriation	12	10 (12.0 %)	12 (0.06)	11 (0.37 %)
	Contusion	8	6 (7.2 %)	8 (0.04)	8 (0.27 %)
Metabolism and nutrition disorders	Vitamin D deficiency	5	5 (6.0 %)	5 (0.03)	5 (0.17 %)
Musculoskeletal and connective tissue disorders	Arthralgia	24	16 (19.3 %)	24 (0.13)	24 (0.81 %)
	Myalgia	49	12 (14.5 %)	49 (0.26)	49 (1.66 %)
	Back pain	14	11 (13.3 %)	14 (0.07)	14 (0.47 %)
	Pain in extremity	13	8 (9.6 %)	13 (0.07)	10 (0.34 %)
Nervous system disorders	Headache	111	45 (54.2 %)	111 (0.59)	104 (3.51 %)
	Dizziness	25	14 (16.9 %)	25 (0.13)	24 (0.81 %)
	Migraine	23	10 (12.0 %)	23 (0.12)	19 (0.64 %)
Psychiatric disorders	Anxiety	9	8 (9.6 %)	9 (0.05)	9 (0.30 %)
	Insomnia	6	6 (7.2 %)	6 (0.03)	6 (0.20 %)
	Depression	5	5 (6.0 %)	5 (0.03)	5 (0.17 %)
Respiratory, thoracic, and mediastinal disorders	Asthma	60	24 (28.9 %)	60 (0.32)	56 (1.89 %)
	Cough	22	16 (19.3 %)	22 (0.12)	21 (0.71 %)
	Nasal congestion	23	11 (13.3 %)	23 (0.12)	23 (0.78 %)
	Oropharyngeal pain	11	8 (9.6 %)	11 (0.06)	11 (0.37 %)
	Epistaxis	17	6 (7.2 %)	17 (0.09)	15 (0.51 %)
	Allergic rhinitis	6	5 (6.0 %)	6 (0.03)	5 (0.17 %)
Skin and subcutaneous tissue disorders	Contact dermatitis	10	8 (9.6 %)	10 (0.05)	10 (0.34 %)
	Rash	8	8 (9.6 %)	8 (0.04)	8 (0.27 %)
	Erythema	7	6 (7.2 %)	7 (0.04)	7 (0.24 %)
	Urticaria	6	6 (7.2 %)	6 (0.03)	6 (0.20 %)
	Pruritus	6	5 (6.0 %)	6 (0.03)	6 (0.20 %)
Vascular disorders	Hypertension	16	12 (14.5 %)	16 (0.09)	14 (0.47 %)

^a^Total number of AEs divided by the total number of subject-years while on IGSC with rHuPH20 treatment

^b^Number of infusions associated with an AE divided by the total number of infusions and presented as a percent (i.e., multiplied by 100)

**Fig. 2:**
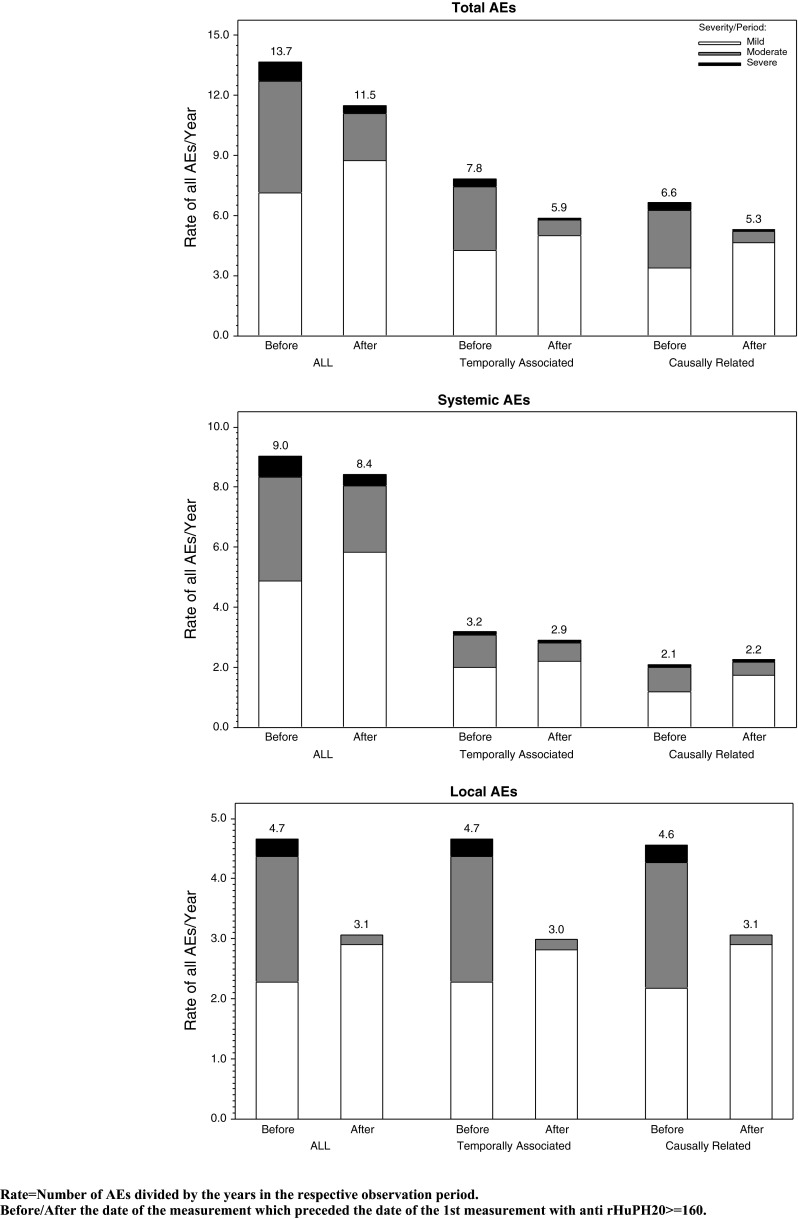
Summary of all, related, or temporally associated AEs (excluding infections) by severity for subjects who developed anti-rHuPH20 antibodies (pivotal study including ramp-up and extension study)

No subject developed neutralizing antibodies to rHuPH20. Low anti-rHuPH20 antibody titers were determined in PIDD subjects including those who, due to their underlying immunodeficiency syndrome, were incapable of producing any type of antibodies. As low anti-rHuPH20 antibody titers were also identified in normal healthy blood donors, titers <1:160 were considered to be consistent with passive transfer from IGSC and therefore ignored in subsequent analyses [26]. However, 13 subjects developed anti-rHuPH20 antibody titers ≥1:160 during the pivotal study. Titers ≥1:160 persisted (i.e., two or more consecutive values) in 6/11 subjects who rolled over into the extension study; another two subjects developed a single increase to 1:160 during the extension study. Anti-rHuPH20 antibody titers typically declined during continued IGHy treatment and thereafter further decreased during the safety follow-up on IGIV or IGSC (data not shown). Annual rates of total, systemic, and local AEs in subjects who developed anti-rHuPH20 antibodies ≥1:160 were similar before and after the first positive titer of anti-rHuPH20 antibodies ≥1:160 (Fig. 2).

### Infections

The annual rate of all infections during IGHy exposure (*N* = 83) was 2.99 (95 % CI 2.60–3.42) per subject. Infection rates per subject-year were comparable between subjects <18 years 3.02 (95 % CI 2.15–4.10) and those ≥18 years 2.98 (95 % CI 2.56–3.44) (Table 3).

**Table 3:** Rate of infections and validated acute serious bacterial infections during IGHy treatment (including ramp-up) by age group (<18, ≥18 years)

Age group^a^ (years)	Number of subjects	Rate of all infections/year		Rate of validated acute serious bacterial infections/year	
		Point estimate	95 % CI	Point estimate	Upper limit of 99 % CI^b^
<18	24	3.02	2.15 to 4.10	0.08	0.20
≥18	59	2.98	2.56 to 3.44	0.01	0.02
Total	83	2.99	2.60 to 3.42	0.03	0.05

^a^Age at screening in pivotal study

^b^Likelihood ratio-based CI on Poisson distribution

A total of five infections, including one that occurred during the IGHy ramp-up period, met the criteria for validated acute serious bacterial infections (VASBIs) [27]. All five VASBIs were pneumonias. The annual rate of VASBIs during IGHy treatment was 0.03 (Table 3).

Common infections during IGHy exposure included sinusitis reported by 56.6 % of subjects followed by upper respiratory tract infection (49.4 %), viral upper respiratory tract infection (21.7 %), and bronchitis (20.5 %) (Table 2).

The rate of infections determined over 1-year periods of IGHy treatment remained stable over the studies, and rates were comparable between subjects <18 years and subjects ≥18 years. As the duration of IGHy treatment varied, the number of subjects exposed had substantially decreased by the final 12-month period shown (months 25 to 36) (Table 4).

**Table 4:** Rate of infections during IGHy treatment^a^ (including ramp-up) in 1-year periods by age group (<18, ≥18 years)

Period	<18 years			≥18 years			All subjects		
		Rate of infections/1-year period			Rate of infections/1-year period			Rate of infections/1-year period	
	Number of subjects	Point estimate	95 % CI	Number of subjects	Point estimate	95 % CI	Number of subjects	Point estimate	95 % CI
Months 1 to 12	18	3.39	2.19 to 4.97	51	3.20	2.54 to 3.96	69	3.25	2.66 to 3.92
Months 7 to 18	13	2.93	1.84 to 4.38	43	2.75	2.15 to 3.44	56	2.79	2.26 to 3.39
Months 13 to 24	13	2.62	1.60 to 4.00	38	3.61	2.84 to 4.50	51	3.36	2.71 to 4.10
Months 19 to 30	12	2.67	1.34 to 4.66	37	3.22	2.49 to 4.07	49	3.08	2.42 to 3.86
Months 25 to 36	7	1.71	0.93 to 2.85	23	2.52	1.69 to 3.58	30	2.33	1.66 to 3.16

^a^ All subjects exposed to IGHy in the pivotal study or in both studies

### Trough Levels

Eighteen subjects infused IGHy at 2-week intervals at some time during the IGHy treatment period (excluding ramp-up). Median steady state trough levels did not substantially vary with the length of the infusion interval and were similar for the 2- and 3-week regimens (1135 mg/dL [95 % CI 939–1440] and 1195 mg/dL [95 % CI 958–1530], respectively) and slightly lower for the 4-week regimen (983 mg/dL [95 % CI 946–1070] (Table 5). At all infusion intervals, trough levels after SC administration of IGHy were substantially above the 500 mg/dL level considered the minimum target for IgG replacement [28, 29].

**Table 5:** Steady-state trough levels^a^ maintained during IGHy treatment (excluding ramp-up) by infusion interval and by age group (<18, ≥18 years)

		IgG trough level (mg/dL)		
Age group^b^(years)	Infusion interval (weeks)	Number of subjects	Median	95 % CI for median
<18	2	2	1660	NA
	3	4	1094	914 to 1520
	4	18	1009	852 to 1200
≥18	2	16	1070	939 to 1330
	3	10	1195	944 to 1580
	4	48	976	935 to 1080
Total	2	18	1135	939 to 1440
	3	14	1195	958 to 1530
	4	66	983	946 to 1070

^a^ An IgG trough level qualifies as steady-state trough level if : for a 4-week infusion interval, the last two infusions; for a 3-week infusion interval, the last three infusions; and for a 2-week infusion interval, the last four infusions were no more than 2 days off the planned infusion date. If more than one IgG trough level qualified, the latest (per infusion interval) was taken

^b^Age at screening in pivotal study

### Specific Antibodies to Relevant Pathogens

No substantial differences were observed between the median levels of antibodies against *Haemophilus influenzae*, hepatitis B surface antigen (HBsAg), and *Clostridium tetani* toxoid at the end of IGIV treatment in the pivotal study and at the end of IGHy treatment in the extension study. For both treatment modalities, the median levels determined were substantially above protective ranges (online supplementary material Table E6 and Table E7).

### Days Off School/Work, in Hospital, and on Antibiotics

Rates per year of 5.75 (95 % CI 4.28–7.52) days off school/work, 4.67 (95 % CI 3.84–5.60) non-study out-patient visits, 0.12 (95 % CI 0.08–0.18) hospitalizations, and 0.61 (95 % CI 0.36–0.94) days in hospital were determined during extended IGHy replacement therapy in PIDD patients. The rate of days receiving antibiotics per year, including brief infection prophylaxis, e.g., for surgery or dental procedures, was 65.39 (95 % CI 48.32–86.09) (online supplementary material Table E8).

### Treatment Preference

Across the pivotal and extension studies, 48/69 (69.6 %) subjects preferred IGHy treatment over alternative modes of treatment. Fifteen of 69 (21.7 %) subjects preferred IV, and 4/69 (5.8 %) subjects preferred regular SC treatment. Twenty-one of 28 (75.0 %) subjects who had received SC treatment before switching to IGHy and 27/41 (65.9 %) subjects previously treated IV indicated their preference for enzyme-facilitated SC infusion (online supplementary material Table E9).

## Discussion

Replacement therapy with IGSC facilitated by rHuPH20 (IGHy) combines the 3- to 4-week infusion frequency of IGIV with the safety, tolerability, and convenience of IGSC treatment of patients with PIDD [23].

The pivotal and subsequent extension study of IGHy reported here spanned one of the longest phase 3 periods of IGSC replacement in PIDD with the greatest IgG exposure reported to date.

During a total of nearly 188 subject-years of IGHy exposure, 4.35 AEs per subject-year (2.60 local and 1.75 systemic AEs per subject-year) were considered related to IGHy by the investigator.

The rate of related systemic AEs over 1-year periods remained consistently low while the rate of related local AEs gradually decreased from 3.68 during months 1–12 to 1.51 per subject-year after 30 months of IGHy treatment, demonstrating that long-term exposure to IGHy did not increase the rate of local AEs. The apparent decrease in local AEs is similar to what has been seen in previous studies of SC IgG replacement therapy [3, 17, 30].

Although the mean IGHy volume of 292.2 mL in a single infusion site was approximately 10- to 15-fold higher [23] and the median maximum infusion rate of 300 mL/h [23] was approximately 10- to 12-fold higher than the typical SC IgG infusion volume and rate [3, 12, 17, 30], rates of local AEs per infusion during IGHy treatment compare favorably with rates reported in previous studies of SC IgG infusion [10, 17, 30]. Observations in a recent analysis of SC IgG replacement patterns in an obese population indicating a lower frequency of local AEs in subjects with a BMI >30 [31] were not confirmed in our study. The rate of local AEs/infusion was low in all BMI groups, and although the proportion of moderate and severe local AEs appeared to be highest in subjects with a BMI >30 and lowest in subjects with a BMI between 25 and 30, differences were small and BMI groups were not coherent in the number of subjects in each group and the length of observation period per subject. Therefore, no firm conclusions on the relationship between rate/severity of local AEs and BMI can be drawn from the data.

Neutralizing antibodies to rHuPH20 were not detected at any time during or after exposure to IGHy. Fifteen of 83 subjects developed anti-rHuPH20 antibody titers ≥1:160 that were not consistent with passive transfer at least once after the first IGHy exposure. All of these subjects had an immunodeficiency disorder that could allow limited antibody formation, especially to protein antigens. The treatment-emergent anti-rHuPH20 antibodies determined in the patients with titers ≥1:160 exhibited an isotype distribution and binding characteristics similar to the antibodies identified in healthy individuals with no exposure to rHuPH20 [26]. Thus, qualitatively, the anti-rHuPH20 antibodies seen in the clinical studies were similar to those seen in normal individuals although the antibody titers were higher in the study subjects. Interestingly, despite continued exposure to rHuPH20, titers had declined substantially by the time IGHy treatment ended, and in the majority of subjects, titers were below 1:160 by the end of the safety follow-up period. Analyses of AEs in subjects with anti-rHuPH20 antibodies showed that AE rates per year were similar before and after the emergence of anti-rHuPH20 antibodies.

The annual rate of all infections during IGHy exposure of 2.99 is comparable to annualized infection rates of 4.5 observed during 3 months of IGIV treatment in the pivotal study [23] and of 4.1 during a median exposure of 379 days to IGSC alone [3]. Infection rates with IGHy were in line also with those reported with other IV [32–34] and SC [10, 17, 30, 35] IgG preparations. In addition, infection rates determined over 1-year periods remained consistent throughout the study, indicating that IGHy remained effective in the prevention of infections during long-term exposure (>49 subjects who received up to 30 months of IGHy treatment). The annual rate of VASBIs during IGHy treatment was 0.03 (upper limit of 99 % CI 0.05), i.e., substantially lower than 1.0 VASBIs/year, which is the threshold specified by regulatory guidance as providing substantial evidence of efficacy [27].

Consistent with the low infection rate, high median steady-state IgG trough levels of 1135, 1195, and 983 mg/dL were attained after SC administration of IGHy at a 2-, 3-, and 4-week schedule, respectively.

Rates per year for days off school/work (5.75), days receiving antibiotics (65.39), and days in hospital (0.61) were well within the range reported for other IV and SC preparations [10, 15, 17, 30, 32–36] and confirmed that replacement therapy with IGHy remained effective during long-term treatment of subjects with PIDD. Irrespective of their previous route of administration, subjects preferred IGHy treatment to IV and SC replacement therapy.

## Conclusions

Long-term replacement therapy with IGHy was safe and effective in pediatric and adult subjects with PIDD. The efficacy of IGHy in preventing infections was maintained over time, and IgG trough levels remained high after long-term exposure. Rates of both systemic and local AEs were low, and the rate of local AEs declined during the IGHy treatment course.

## Electronic supplementary material

Below is the link to the electronic supplementary material.

ESM 1 (DOC 178 kb)
