# Supplementary material for: Long-Term Tolerability, Safety, and Efficacy of Recombinant Human Hyaluronidase-Facilitated Subcutaneous Infusion of Human Immunoglobulin for Primary Immunodeficiency
Source: J Clin Immunol. 2016 May 25;36:571–82. doi: 10.1007/s10875-016-0298-x (PMC4940441; doi:10.1007/s10875-016-0298-x)
Supplement: Supplementary file 1 — (DOC 178 kb) [file 10875_2016_298_MOESM1_ESM.doc]

Journal of Clinical Immunology - Online Supplementary Materials for:

**Long-term Tolerability, Safety and Efficacy of Recombinant Human Hyaluronidase-facilitated Subcutaneous Infusion of Human Immunoglobulin for Primary Immunodeficiency**

Richard L. Wasserman, MD, PhD,^a^ Isaac Melamed, MD,^b^ Mark R. Stein, MD,^c^ Werner Engl, PhD,^d^ Marlies Sharkhawy, MA,^d^ Heinz Leibl, PhD,^d^ Jennifer Puck, MD,^e^ Arye Rubinstein, MD,^f^ Lisa Kobrynski, MD,^g^ Sudhir Gupta, MD,^h^ J. Andrew Grant, MD,^i^ Anoshie Ratnayake, MD, MPH,^j^ G. Wendell Richmond, MD,^k^ Joseph Church, MD,^l^ Leman Yel, MD,^m^ David Gelmont, MD^n*^

^a^Allergy Partners of North Texas Research, Dallas, TX; ^b^IMMUNOe Clinical Research Center, Centennial, CO; ^c^Allergy Associates of the Palm Beaches, North Palm Beach, FL; ^d^Baxalta Innovations GmbH, Vienna, Austria; ^e^University of California San Francisco, San Francisco, CA; ^f^Allergy & Immunology Division, Montefiore Medical Center, Bronx, NY; ^g^Emory Children’s Center, Emory University, Atlanta, GA; ^h^University of California, Irvine, CA; ^i^University of Texas Medical Branch, Galveston, TX; ^j^West Coast Clinical Trials, Cypress, CA; ^k^Allergy and Asthma Physicians, Hinsdale, IL; ^l^ Children’s Hospital Los Angeles, Los Angeles, CA; ^m^Baxalta US Inc., Cambridge, MA;  ^n^Baxalta US Inc., Westlake Village, CA

*Corresponding Author: David Gelmont, MD, Baxalta US Inc., One Baxter Way, Westlake Village, CA 91362-3811
e-mail: [david.gelmont@baxalta.com](mailto:david.gelmont@baxalta.com)

| Table E1 Demographic Characteristics at Screening by Age Group (<18, ≥18 Years) | | | | |
| --- | --- | --- | --- | --- |
| **Categorical Data** | | | | |
| **Parameters^a^** | **Category** | **Subjects  <18 Years N=26 n (%)** | **Subjects  ≥18 Years N=63 n (%)** | **Total N=89 n (%)** |
| Gender | Male | 15 (57.7) | 31 (49.2) | 46 (51.7) |
|  | Female | 11 (42.3) | 32 (50.8) | 43 (48.3) |
| Race | White | 25 (96.2) | 56 (88.9) | 81 (91.0) |
|  | Black or African American | 1 (3.8) | 1 (1.6) | 2 (2.2) |
|  | Asian | 0 | 3 (4.8) | 3 (3.4) |
|  | American Indian or Alaska Native | 0 | 1 (1.6) | 1 (1.1) |
|  | Multiple | 0 | 2 (3.2) | 2 (2.2) |
| Ethnicity | Hispanic or Latino | 3 (11.5) | 5 (7.9) | 8 (9.0) |
|  | Not Hispanic or Latino | 23 (88.5) | 58 (92.1) | 81 (91.0) |
| **Continuous Data** | | | | |
| **Parameters^a^** | **Statistics** | **Subjects  <18 Years** | **Subjects  ≥18 Years** | **Total** |
| Age [years] | N | 26 | 63 | 89 |
|  | Min | 4 | 18 | 4 |
|  | Median | 11.0 | 49.0 | 36.0 |
|  | Max | 17 | 78 | 78 |
| Height [cm] | N | 26 | 63 | 89 |
|  | Min | 94 | 108 | 94 |
|  | Median | 145.0 | 167.6 | 165.0 |
|  | Max | 175 | 193 | 193 |
| Weight [kg] | N | 26 | 63 | 89 |
|  | Min | 15 | 45 | 15 |
|  | Median | 42.0 | 70.7 | 63.8 |
|  | Max | 117 | 136 | 136 |
| ^a^ At screening during first study of participation | | | | |

| Table E2  Number and Rate of Adverse Events (Excluding Infections) per Subject-year During IGHy Treatment  (Including Ramp-up)  by Age Group (<18, ≥18 Years) | | | | | | | |
| --- | --- | --- | --- | --- | --- | --- | --- |
|  |  | Local AEs Number and Rate per Subject-year^a^ | | | Systemic AEs  Number and Rate per Subject-year^a^ | | |
| Age Group^b^ (years) | Total Number of Subject-years of Treatment^c^ | Total | Temporally Associated^d^ | Related^e^ | Total | Temporally Associated^d^ | Related^e^ |
| <18 | 48.66 | 69 (1.42) | 69 (1.42) | 67 (1.38) | 307 (6.31) | 98 (2.01) | 70 (1.44) |
| ≥18 | 139.04 | 429 (3.09) | 419 (3.01) | 421 (3.03) | 1200 (8.63) | 393 (2.83) | 259 (1.86) |
| Total | 187.69 | 498 (2.65) | 488 (2.60) | 488 (2.60) | 1507 (8.03) | 491 (2.62) | 329 (1.75) |
| ^a^ Total number of adverse events (AEs) divided by the total number of subject-years on IGHy treatment ^b^ Age at screening in pivotal study ^c^ Subject-years of treatment = One-year period on IGHy  ^d^ During or within 72 h after treatment ^e^ Related to IGSC and/or rHuPH20. | | | | | | | |

| **Table E3  Rate of Adverse Events (Excluding Infections) per Subject-year During IGHy Treatment  (Including Ramp-up)  in One-year Periods** | | | | | | | |
| --- | --- | --- | --- | --- | --- | --- | --- |
|  | | **Local AEs  Rate per Subject-year^a^** | | | **Systemic AEs  Rate per Subject-year^a^** | | |
| **Period  (# of Infusions)** | **Number of Subjects-years of Treatment^b^** | **Total** | **Temporally Associated^c^** | **Related^d^** | **Total** | **Temporally Associated^c^** | **Related^d^** |
| Month 1 to 12 (1086) | 69 | 3.79 | 3.70 | 3.68 | 7.86 | 2.74 | 1.94 |
| Month 7 to 18 (774) | 56 | 2.64 | 2.57 | 2.57 | 6.84 | 2.16 | 1.47 |
| Month 13 to 24 (778) | 51 | 2.16 | 2.14 | 2.12 | 7.28 | 2.04 | 1.24 |
| Month 19 to 30 (833) | 49 | 1.51 | 1.51 | 1.51 | 8.22 | 2.33 | 1.41 |
| Month 25 to 36 (539) | 30 | 0.37 | 0.37 | 0.37 | 7.98 | 1.70 | 0.63 |
| ^a^ Total number of adverse events (AEs) divided by the total number of subject-years while on IGSC with rHuPH20 treatment. ^b^ Subject-years of treatment = One-year period on IGSC with rHuPH20 ^c^ During or within 72 h after treatment ^d^ Related to IGSC and/or rHuPH20. | | | | | | | |

| **Table E4 Local Adverse Events (Including Infections) During IGHy Treatment (Including Ramp-up)  Categorized by Treatment, Body Mass Index and Severity** | | | | | | | |
| --- | --- | --- | --- | --- | --- | --- | --- |
| **BMI^a^ Group** | **Severity** | **Total Number of AEs** | **Total Number of Subjects** | **Total Number of Infusions** | **Rate per Subject^b^** | **Rate per Infusion^c^** | **Subjects Experiencing AE, n (%^d^)** |
| BMI < 25 | Mild | 156 | 48 | 1547 | 3.25 | 0.10 | 33 (68.8) |
|  | Moderate | 47 | 48 | 1547 | 0.98 | 0.03 | 14 (29.2) |
|  | Severe | 2 | 48 | 1547 | 0.04 | 0.00 | 2 (4.2) |
|  | Total | 205 | 48 | 1547 | 4.27 | 0.13 | 35 (72.9) |
| BMI 25-30 | Mild | 115 | 17 | 671 | 6.77 | 0.17 | 11 (64.7) |
|  | Moderate | 12 | 17 | 671 | 0.71 | 0.02 | 4 (23.5) |
|  | Severe | 0 | 17 | 671 | 0.00 | 0.00 | 0 (0.0) |
|  | Total | 127 | 17 | 671 | 7.47 | 0.19 | 11 (64.7) |
| BMI > 30 | Mild | 127 | 18 | 741 | 7.06 | 0.17 | 11 (61.1) |
|  | Moderate | 39 | 18 | 741 | 2.17 | 0.05 | 10 (55.6) |
|  | Severe | 2 | 18 | 741 | 0.11 | 0.00 | 1 (5.6) |
|  | Total | 168 | 18 | 741 | 9.33 | 0.23 | 14 (77.8) |
| Total | Mild | 398 | 83 | 2959 | 4.80 | 0.14 | 55 (66.3) |
|  | Moderate | 98 | 83 | 2959 | 1.18 | 0.03 | 28 (33.7) |
|  | Severe | 4 | 83 | 2959 | 0.05 | 0.00 | 3 (3.6) |
|  | Total | 500 | 83 | 2959 | 6.02 | 0.17 | 60 (72.3) |
| ^a^ Body mass index (BMI) at baseline in the pivotal study ^b^ Number of adverse events (AEs) divided by total number of subjects ^c^ Number of adverse events (AEs) divided by total number of infusions ^d^ Number of subjects reporting an adverse event (AE) divided by total number of subjects | | | | | | | |

| **Table E5 Number and Rate of Serious Adverse Events (Excluding Infections) per Subject-year During IGHy Treatment (Including Ramp-up) by Age Group (<18, ≥18 Years)** | | | | | | | |
| --- | --- | --- | --- | --- | --- | --- | --- |
|  | | **Local SAEs  Number and Rate per Subject-year^a^** | | | **Systemic SAEs  Number and Rate per Subject-year^a^** | | |
| **Age Group^b^ (years)** | **Total Number of Subject-years of Treatment^c^** | **Total** | **Temporally Associated^d^** | **Related^e^** | **Total** | **Temporally Associated^d^** | **Related^e^** |
| <18 | 48.66 | 0 (0.00) | 0 (0.00) | 0 (0.00) | 5 (0.10) | 0 (0.00) | 0 (0.00) |
| ≥18 | 139.04 | 0 (0.00) | 0 (0.00) | 0 (0.00) | 18 (0.13) | 3 (0.02) | 0 (0.00) |
| Total | 187.69 | 0 (0.00) | 0 (0.00) | 0 (0.00) | 23 (0.12) | 3 (0.02) | 0 (0.00) |
| ^a^ Total number of serious adverse events (SAEs) divided by the total number of subject-years while on IGHy treatment. ^b^ Age at screening in pivotal study ^c^ Subject-years of treatment = One-year period on IGHy  ^d^ During or within 72 h after treatment ^e^ Related to IGSC and/or rHuPH20 | | | | | | | |

| **Table E6  Specific Antibodies to Haemophilus influenzae and Hepatitis B  During IGHy Treatment  by Age Group (<18, ≥18 Years)** | | | | | |
| --- | --- | --- | --- | --- | --- |
| **Age Group^a^ (years)** | **Pathogen** | **Time Point** | **N** | **Median** | **95% CI for Median** |
| <18 | Haemophilus influenzae [µg/mL] | End of IV in Pivotal Study | 12 | 2.32 | 1.65 to 4.22 |
|  |  | End of IGHy in Pivotal Study | 21 | 2.34 | 1.69 to 2.91 |
|  |  | Baseline of Extension Study | 14 | 2.37 | 1.89 to 4.58 |
|  |  | End of IGHy in Extension Study | 13 | 2.56 | 2.01 to 5.26 |
|  | Hepatitis B [mIU/mL] | End of IV in Pivotal Study | 11 | 168.0 | 126.5 to 291.5 |
|  |  | End of IGHy in Pivotal Study | 21 | 186.7 | 164.6 to 276.7 |
|  |  | Baseline of Extension Study | 15 | 172.5 | 142.1 to 234.6 |
|  |  | End of IGHy in Extension Study | 13 | 291.1 | 208.1 to 387.9 |
| ≥18 | Haemophilus influenzae [µg/mL] | End of IV in Pivotal Study | 21 | 2.43 | 2.05 to 3.06 |
|  |  | End of IGHy in Pivotal Study | 59 | 2.62 | 2.30 to 3.12 |
|  |  | Baseline of Extension Study | 46 | 2.15 | 2.02 to 2.59 |
|  |  | End of IGHy in Extension Study | 44 | 2.82 | 2.41 to 3.21 |
|  | Hepatitis B [mIU/mL] | End of IV in Pivotal Study | 21 | 233.9 | 190.3 to 290.9 |
|  |  | End of IGHy in Pivotal Study | 59 | 259.2 | 234.5 to 316.3 |
|  |  | Baseline of Extension Study | 51 | 189.7 | 171.0 to 232.5 |
|  |  | End of IGHy in Extension Study | 44 | 296.8 | 257.1 to 364.2 |
| Total | Haemophilus influenzae [µg/mL] | End of IV in Pivotal Study | 33 | 2.43 | 2.05 to 3.06 |
|  |  | End of IGHy in Pivotal Study | 80 | 2.54 | 2.30 to 2.93 |
|  |  | Baseline of Extension Study | 60 | 2.16 | 2.03 to 2.59 |
|  |  | End of IGHy in Extension Study | 57 | 2.72 | 2.53 to 3.21 |
|  | Hepatitis B [mIU/mL] | End of IV in Pivotal Study | 32 | 216.1 | 183.8 to 264.3 |
|  |  | End of IGHy in Pivotal Study | 80 | 249.2 | 225.0 to 289.3 |
|  |  | Baseline of Extension Study | 66 | 189.4 | 171.0 to 226.2 |
|  |  | End of IGHy in Extension Study | 57 | 295.4 | 262.2 to 344.3 |
| ^a^ Age at screening in pivotal study | | | | | |

| **Table E7  Anti-Tetanus Antibody [IU/mL] During IGHy Treatment  by Age Group (<18, ≥18 Years)** | | | | |
| --- | --- | --- | --- | --- |
| **Age Group^a^ (years)** | **Time Point** | **N** | **Median** | **95% CI for Median** |
| <18 | End of IV in Pivotal Study | 12 | 2.175 | 1.470 to 2.560 |
|  | End of IGHy in Pivotal Study | 21 | 1.960 | 1.710 to 2.850 |
|  | Baseline of Extension Study | 15 | 1.890 | 1.570 to 2.280 |
|  | End of IGHy in Extension Study | 13 | 2.120 | 1.520 to 2.950 |
| ≥18 | End of IV in Pivotal Study | 21 | 2.320 | 1.800 to 2.800 |
|  | End of IGHy in Pivotal Study | 59 | 2.700 | 2.290 to 3.300 |
|  | Baseline of Extension Study | 47 | 2.310 | 2.040 to 2.840 |
|  | End of IGHy in Extension Study | 44 | 2.905 | 2.370 to 3.590 |
| Total | End of IV in Pivotal Study | 33 | 2.300 | 1.810 to 2.690 |
|  | End of IGHy in Pivotal Study | 80 | 2.550 | 2.280 to 2.850 |
|  | Baseline of Extension Study | 62 | 2.255 | 2.030 to 2.440 |
|  | End of IGHy in Extension Study | 57 | 2.600 | 2.350 to 3.210 |
| ^a^ Age at screening in pivotal study | | | | |

| Table E8  Analysis of Days Off School/Work, on Antibiotics, in Hospital and  Number of Acute Physician Visits and Hospitalizations  During IGHy Treatment^a^ (Including Ramp-up) | | |
| --- | --- | --- |
|  | Rate per Year N=83 | |
| Parameter | Point Estimate | 95% CI |
| Days off school/work | 5.75 | 4.28 to 7.52 |
| Days on antibiotics | 65.39 | 48.32 to 86.09 |
| Number of non-study out-patient visits | 4.67 | 3.84 to 5.60 |
| Number of hospitalizations | 0.12 | 0.08 to 0.18 |
| Days in hospital | 0.61 | 0.36 to 0.94 |
| ^a^ All patients exposed to IGHy in the pivotal study or in both studies. | | |

| Table E9  Subject Preference for IGHy, IGSC, or IGIV | | | |
| --- | --- | --- | --- |
|  | Route of administration before first use of rHuPH20 | | |
| Answer | IV n (%) | SC n (%) | Total n (%) |
| No preference | 1 (2.4%) | 1 (3.6%) | 2 (2.9%) |
| Intravenous administration | 12 (29.3%) | 3 (10.7%) | 15 (21.7%) |
| Regular subcutaneous administration | 1 (2.4%) | 3 (10.7%) | 4 (5.8%) |
| IGHy administration | 27 (65.9%) | 21 (75.0%) | 48 (69.6%) |
| Total | 41 (100.0%) | 28 (100.0%) | 69 (100.0%) |
